# DBDAA: A real-time approach to Dynamic Banker’s Deadlock Avoidance Algorithm with optimized time complexity

**DOI:** 10.1371/journal.pone.0310807

**Published:** 2024-09-20

**Authors:** Most. Fatematuz Zohora, Fahiba Farhin, M. Shamim Kaiser

**Affiliations:** 1 Computer Science and Engineering, Bangladesh Army International University of Science and Technology, Cumilla, Bangladesh; 2 Computer Science and Engineering, International University of Business Agriculture and Technology, Dhaka, Bangladesh; 3 Institute of Information Technology, Jahangirnagar University, Dhaka, Bangladesh; Thamar University: Dhamar University, YEMEN

## Abstract

Effective resource allocation is crucial in operating systems to prevent deadlocks, especially when resources are limited and non-shareable. Traditional methods like the Banker’s algorithm provide solutions but suffer from limitations such as static process handling, high time complexity, and a lack of real-time adaptability. To address these challenges, we propose the Dynamic Banker’s Deadlock Avoidance Algorithm (DBDAA). The DBDAA introduces real-time processing for safety checks, significantly improving system efficiency and reducing the risk of deadlocks. Unlike conventional methods, the DBDAA dynamically includes processes in safety checks, considerably decreasing the number of comparisons required to determine safe states. This optimization reduces the time complexity to O(n) in the best-case and O(*nd*) in the average and worst-case scenarios, compared to the O(*n*^2^*d*) complexity of the original Banker’s algorithm. The integration of real-time processing ensures that all processes can immediately engage in safety checks, improving system responsiveness and making the DBDAA suitable for dynamic and time-sensitive applications. Additionally, the DBDAA introduces a primary unsafe sequence mechanism that enhances the acceptability and efficiency of the algorithm by allowing processes to participate in safety checks repeatedly after a predetermined amount of system-defined time. Experimental comparisons with existing algorithms demonstrate the superiority of the DBDAA in terms of reduced safe state prediction time and increased efficiency, making it a robust solution for deadlock avoidance in real-time systems.

## Introduction

**Fig 1:**
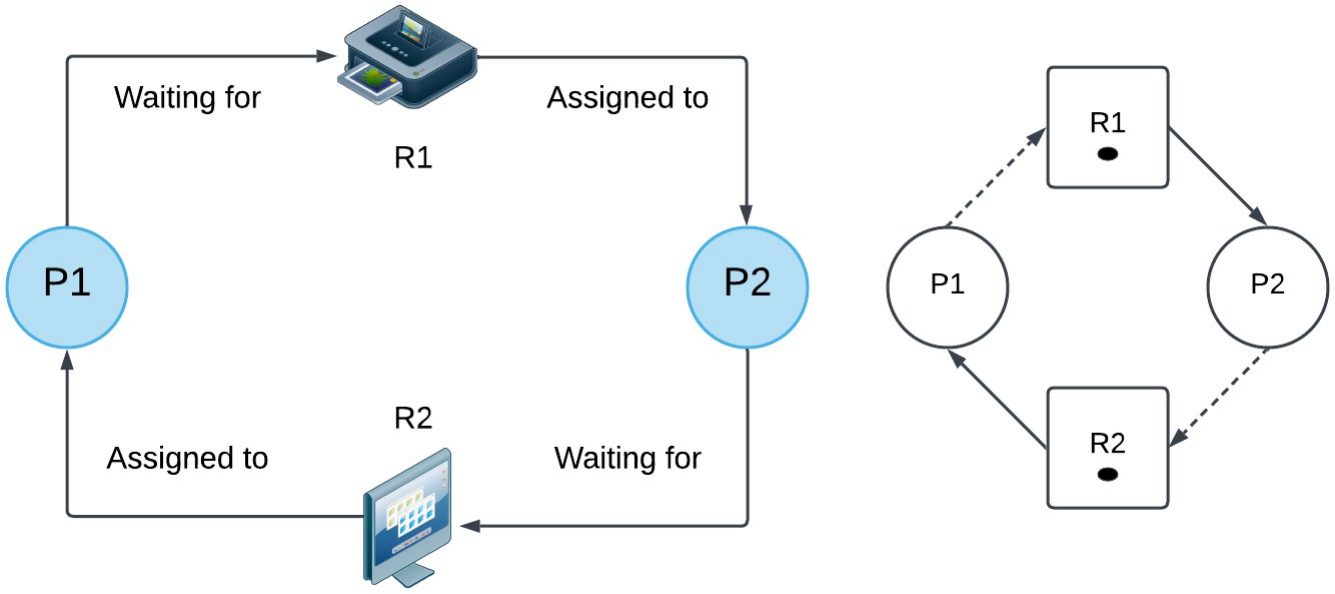
Process execution halted due to deadlock.

In the realm of operating systems and concurrent computing, resource allocation is a critical challenge. A fundamental issue in this area is the occurrence of deadlocks, which happen when a set of processes become indefinitely blocked, each waiting for resources held by the others. Deadlocks lead to system inefficiencies and can cause significant disruptions in computing environments. They occur when four necessary conditions—mutual exclusion, hold and wait, no preemption, and circular wait—are simultaneously present. Addressing deadlocks is essential for ensuring the smooth operation of systems that handle multiple processes and shared resources. Fig 1 demonstrates the basic deadlock scenario situation.

One of the most well-known solutions for deadlock avoidance is Dijkstra’s Banker’s Algorithm [1]. This algorithm functions similarly to a bank lending money and ensures that resources are allocated only if the system remains in a safe state, where no deadlocks can occur. The algorithm requires each process to declare its maximum resource needs upfront, and resources are granted only if they do not lead to an unsafe state. While effective in certain controlled environments, the traditional Banker’s algorithm has significant limitations. Here are a few restrictions:

- Since most systems include a dynamic number of processes, it is impractical to believe that the number of processes is static. The system can not handle the processes in real time as it works on only the reserved processes.
- Banker’s Algorithm requires *n*^2^*d* time complexity in its average and worst case.
- It must be aware of the maximum amount of each resource a process may request. So it may be unable to apply the Banker’s algorithm in maximum cases.
- Furthermore, while a process must eventually release all of its resources (when the process ends) for the algorithm to be valid, a real system does not require this requirement.
- Waiting for hours (or even days) for resources to be released is usually unacceptable.
- It does not give any information when the system is not in a safe state. It does not give details about the process that failed during the execution and also does not give information about the reason why it was not able to give the appropriate safe sequence.

Existing research has faced several challenges, which include high computational overhead due to the large number of comparisons required to predict safe states, prolonged prediction times, and inefficiencies in handling real-time processing demands. To address these issues, this paper introduces a novel approach, the Dynamic Banker’s Deadlock Avoidance Algorithm (DBDAA), designed to address the limitations of static resource allocation mechanisms. Unlike traditional algorithms that rely on a predefined number of processes, the DBDAA dynamically adds processes to the system for safety checks in a real-time manner without any delay. This adaptive methodology not only enhances system performance but also significantly reduces the risk of deadlocks in highly variable operational contexts. To summarize, our research makes the following contributions:

1. **Reduced Number of Comparisons and Decreased Prediction Time:** The proposed algorithm significantly reduces the number of comparisons needed to predict the safe state of a system. By minimizing the number of security checks required to find the safe state, the overall time complexity is reduced to *O*(*nd*), compared to the original Banker’s algorithm which has a time complexity of *O*(*n*^2^*d*). This optimization of the prediction process results in faster prediction times and improved system performance.
2. **Increased Acceptability and Efficiency:** The algorithm introduces the concept of a primary unsafe sequence, which temporarily holds unsafe sequences. This mechanism allows processes to participate in safety checks when additional resource instances become available, thereby increasing the algorithm’s acceptability and efficiency.
3. **Real-Time Processing:** The algorithm enhances real-time processing by allowing all processes to participate in safety checks by adding themselves directly to the ready queue, rather than waiting for the current computation to complete. This approach ensures efficiency, reduces computational overhead, and makes the algorithm suitable for dynamic and time-sensitive applications.

The rest of this paper is organized as follows: The Related Work section covers a literature review and the limitations of existing deadlock avoidance algorithms. The Methodology section provides details on the theoretical foundations and implementation of the DBDAA with a series of experiments and simulations to evaluate the performance of the proposed algorithm. The Result and Discussion section discusses the results and implications of our findings with the comparisons with existing research. Finally, the Conclusion and Future Works section concludes the paper and suggests directions for future research.

## Related work

Recent advancements in deadlock avoidance algorithms have focused on improving the efficiency of the classic Banker’s algorithm. Dixit *etal*. introduced a dynamic and improved implementation of the Banker’s Algorithm. This approach allows for resource changes at runtime and provides detailed resource and process requirements, significantly enhancing the algorithm’s flexibility and efficiency. Their experimental results demonstrated a 9% improvement in performance over the original Banker’s Algorithm, particularly in reducing execution time and improving deadlock detection. The study simulated scenarios involving five processes and three types of resources. However, it did not fully address scenarios where the system is already in an unsafe state, nor did it comprehensively validate dynamic processes [2].

A notable contribution in this area is a new algorithm that builds upon the foundational principles of the Banker’s algorithm but introduces significant optimisations. Momotaz *et. al*. proposed an algorithm that demonstrates remarkable improvements in both time and space complexities. It eliminates the need for a complex permutation matrix and utilises dynamic memory allocation, reducing memory requirements. Experimental results show that this algorithm is n times faster than the original Banker’s algorithm and d times faster than some contemporary modifications, such as the one proposed in compared studies [3]. In the best-case scenario, it requires only n comparisons to detect the safe state of any system, compared to the O(nd) comparisons needed by the original algorithm, making it more efficient as the number of resource types increases. This advancement underscores the potential for more robust and scalable solutions in deadlock avoidance. Though it can perform better in the best case, in the worst and average case it produces an unsafe sequence whereas traditional bankers can provide solutions [4].

Bondarenko and Azeez presented a modified approach to the Banker’s algorithm aimed at enhancing resource allocation efficiency in cloud environments. Their method checks that the system is in a safe condition and is not in a deadlock by using the attribute of execution time of the process to obtain better resource allocation. Additionally, their approach utilizes a systematic evaluation of resource requests and allocation sequences to ensure safety and avoid deadlocks. Despite these advancements, the algorithm still faces limitations, including substantial execution time and computational complexity, which can hinder its performance in real-time processing scenarios [5].

Song *et. al*. proposed modified algorithm which addresses the limitations associated with the high costs of time complexity, space complexity, and restricted concurrency inherent in the traditional Banker’s Algorithm. By allowing processes to dynamically release resources during their execution rather than holding all resources until termination, the modified algorithm significantly enhances system resource utilization and concurrency. Experimental results indicate that this method reduces the total cost time by approximately 10% compared to the original Banker’s Algorithm and performs better than compared researches [2, 6–8]. This improvement demonstrates the potential for more efficient resource allocation and deadlock avoidance in various computing environments [9].

In recent times, Wicaksono *et. al*. introduced a new algorithm that presents an improved version of the existing banker’s method for dynamically avoiding deadlocks in the operating system. When a new process initiates a new resource request in the middle of the banker’s algorithm execution, it must wait until the banker’s algorithm returns a value before beginning another with fresh data. The banker’s algorithm has been improved for this situation. In the middle of the banker’s algorithm execution, a new process can intercept it and inject a new resource request, after which the execution will continue to check and generate a safe sequence. The security sequence ensures that the operating system avoids deadlocks. This approach prevents deadlocks and improves system performance, as demonstrated by simulation and experimental testing [10]. The new algorithm is notably faster than other recent modifications [9, 11]. This makes it particularly effective as the number of resource types increases, thereby enhancing prediction time and enabling real-time processing [12].

In addition to advancements in deadlock avoidance and resource allocation within operating systems, similar optimization challenges are being addressed in other domains, such as medical diagnostics. Techniques like hybrid neural networks combined with optimization algorithms have been applied to enhance the accuracy and efficiency of disease detection, including melanoma detection, skin cancer diagnosis, and kidney stone identification [13–16]. These approaches highlight the broader applicability of optimization strategies in managing complex resource allocation tasks across various fields.

Too many uses of traditional Banker’s algorithms for different services are common in various research studies. Samha considers the most recent use of cloud computing in research [17]. This research proposes a novel IaaS cloud framework emphasising virtualisation, including virtual machine migration and resource consolidation. It incorporates Trust Manager (TM) and Broker Manager (BM) components for enhanced SLA monitoring and trust evaluation. The methodology includes user profiling and advanced ranking algorithms. The use of the Banker’s algorithm and a comprehensive Service Level Agreement (SLA) Management plan provides efficient resource allocation here.

Besides resource allocation, scheduling algorithms have also seen enhancements in cloud computing environments. A notable example is the enhanced round-robin algorithm that utilizes a dynamic time quantum to manage real-time asymmetric burst length processes. This approach improves system responsiveness and resource utilization, making it highly effective in dynamic cloud environments where workload characteristics can vary significantly [18].

A summary of the highlighted work done in this field is given in Table 1:

**Table 1:** Summary of related studies.

Ref	Contributions	Result	Dataset Consideration	Remarks
[2]	Introduced a dynamic approach to the Banker’s Algorithm, allowing resource changes at runtime and providing detailed resource and process requirements	reduced execution time and improved deadlock detection, with an approximate 9% performance improvement over the original Banker’s Algorithm	Simulated with 5 processes and 3 resource types	Does not address scenarios where the system is already in an unsafe state; dynamic processes not fully validated
[4]	Proposed algorithm uses sorting and linked lists to streamline safety detection by prioritizing processes with the lowest resource needs and simplifying comparisons	Reduced time complexity to *O*(*nd*) and space complexity below the original Banker’s algorithm at the time of finding safe state	Simulated with 5 resources with 10 processes	Work better for best case, but provide wrong answer in several cases
[5]	Eliminated permutation matrices; used dynamic memory allocation; evaluated resource requests systematically	Algorithm is n times faster than original and d times faster than contemporary modifications.	Simulated with 10 resource types and 20 processes.	High execution time and computational complexity, limiting real-time processing.
[9]	Proposed a modified banker’s algorithm that dynamically releases resources during process execution, improving resource utilization in the Reducing	Reduced total cost time by 10–13% compared to the original, particularly when releasing resources, while maintaining or improving performance as resource quantity increases.	Simulated with resources 20 and 30, with 2 releases	Limited to simulation; static process consideration only
[12]	Introduced an optimized version of the banker’s algorithm that dynamically adjusts the Need[M, N] matrix based on new resource requests, recalculating before each loop execution	Successfully generated safe sequences, allowing real-time resource allocation	Simulated with 5 processes and 3 resource types on Python (Windows 11)	Limited to simulation; lacks validation in real-world applications; no consideration of time complexity
[17]	Proposed a novel IaaS cloud framework with VM migration and resource consolidation, incorporating Trust Manager and Broker Manager components; Used Banker’s algorithm as a part of resource allocation	Improved cloud service security, trust, and resource management efficiency	Simulated with 100 VMs and 50 physical machines	May not cover all configurations in federated cloud environments; real-world performance impact not addressed

## Methodology

The sorting mechanism and linked list data structure provide the basis for our suggested approach. The approach organizes processes in ascending order according to the maximum number of resources (any type) needed by each process. Consequently, the processes with the lowest resource requirements appear first when we evaluate the processes in the safety algorithm. A process is checked once in a specific amount of time. Our approach lowers the complexity of the safety detecting phase by employing the sorting method. A detailed explanation of the proposed DBDAA is provided below:

### Data structures and definitions

This subsection presents the data structures and abbreviations used in DBDAA. Let,

*n* = number of processes,

*P*_*i*_ = *i*^*th*^ number process,

*R*_*j*_ = *j*^*th*^ number resource,

*d* = The number of resources type,

*m* = One cycle,

*AT*_*i*_ = Arrival Time of *i*^*th*^ number process,

*BT*_*i*_ = Burst Time of *i*^*th*^ number process,

*RQ* = Ready Queue,

*SRQ* = Sorted Ready Queue,

*Available*[*j*] = The number of *R*_*j*_ resources available in the system,

*Maximum*[*i*, *j*] = Maximum demand quantity of *R*_*j*_ resources for process *P*_*i*_,

*Allocation*[*i*, *j*] = The number of *R*_*j*_ resources obtained by process *P*_*i*_,

*Need*[*i*, *j*] = The number of *R*_*j*_ resources required for the process *P*_*i*_,

*Max*_*Need* = The Maximum value of the *Need*[*i*, *j*] for each process,

*Min*_*A* = The minimum available resource from *Available*[*j*],

*Safe*_*Sequence* = An order of processes for execution avoiding deadlock,

*Primary*_*Unsafe*_*Sequence* = An order of processes that are unsafe to execute currently,

*Unsafe*_*Sequence* = An order of processes that are completely unsafe for the system.

### Proposed algorithm

In our proposed algorithm [Algorithm 1], Dynamic Banker’s Deadlock Avoidance Algorithm (DBDAA) allows processes to join the system in a real-time manner and take part in the safety check procedure instantly without any delay. A detailed, step-by-step explanation of the algorithm is given below:

1. Input Resources: The algorithm starts by taking input for each resource type *d* of the system as *Resource*[*j*], where j = 1…*d*. Initialize a temporary variable *Available*[*j*] = *Resource*[*j*].
2. Set Timer: A timer defining a cycle for a safety check is set to the current time plus *m* units.
3. Initialize Process: New process *P*_*i*_ is initialized with its arrival time (*AT*[*i*]), burst time (*BT*[*i*]), maximum resource needs (*Maximum*[*i*, *j*]), and current resource allocation (*Allocation*[*i*, *j*]).
4. Check Resource Availability: If the maximum resource need *Maximum*[*i*, *j*] for any process exceeds the available resources *Resource*[*j*], the process is marked as unsafe and added to the *Unsafe*_*Sequence* list. Otherwise, the process is set as incomplete and added to the Ready Queue (RQ) and new *Available*[*j*] is calculated as *Available*[*j*]—*Allocation*[*i*, *j*]. Then we calculate the need for each process as *Need*[*i*, *j*] = *Maximum*[*i*, *j*] − *Allocation*[*i*, *j*] and also determine the maximum need as *Max*_*Need* for each process.
5. Sort Ready Queue: The Ready Queue (RQ) is sorted in ascending order based on the *Max*_*Need* of the processes, forming the Sorted Ready Queue (*SRQ*).
6. Safety Check: While the processor is free and the *SRQ* is not empty the first process *P*_*i*_ from the *SRQ* is selected and initialized counter variable, k = 1.
  - If k is less than or equal to half the total number of resources *d*, for all unmarked resources identify the maximum need *Max*_*Need* from *Need*[*i*, *j*] and the minimum available resource *Min*_*A* from *Available*[*j*]. The process is in a safe state if *Max*_*Need* is less than or equal to *Min*_*A*. Otherwise, locate the positions of the *Max*_*Need* (L1) and *Min*_*A* (L2). The process is added to the *Primary*_*Unsafe*_*Sequence* and the algorithm jumps to the timer check step if *Need*[*i*, *L*1] exceeds *Available*[*L*1] or *Need*[*i*, *L*2] exceeds *Available*[*L*2]. If not, increment k and mark the resources *Available*[*L*1] and *Available*[*L*2] before rechecking.
  - If k is greater than half the total number of resources *d* and all available resource types are marked, proceed to complete the process step. If a resource is still unmarked, find its location as L3 and check the need for the unmarked resources by process *P*_*i*_. If *Need*[*i*, *L*3] exceeds *Available*[*L*3], the process is added to the *Primary*_*Unsafe*_*Sequence*, and the algorithm jumps to the timer Check step. Otherwise, proceed to complete the process step.
7. Complete Process: We mark the process as complete and add it to the *Safe*_*Sequence* list. Update the available resources *Available*[*j*] by adding the allocated resources of the process *Allocation*[*i*, *j*] back and unmark all resource types.
8. Timer Check: Every time the system adds a process to the *Safe*_*Sequence* or the *Primary*_*Unsafe*_*Sequence*, it checks the *timer*. If the current time reaches the *timer* value or the *SRQ* is empty the system returns the *Safe*_*Sequence*, *Primary*_*Unsafe*_*Sequence*, and *Unsafe*_*Sequence*. All processes in the *Primary*_*Unsafe*_*Sequence* are added back to the *SRQ* and continue from the safety check step. Otherwise, the system carries out its safety check process.
9. Wait State: If no processes are in the *SRQ* or the processor is busy, the system waits.

**Algorithm 1** The DBDAA Algorithm

1: Input *d* numbers of resources, *Resource*[*j*]

2: set *Available*[*j*] = *Resource*[*j*] and *Timer* = Current_time + *m* unit

3: Initialize new Process *P*_*i*_ with *AT*[*i*], *BT*[*i*], *Maximum*[*i*, *j*], *Allocation*[*i*, *j*]

4: **if** *Maximum*[*i*, *j*] > *Resource*[*j*] **then**

5:  Push *P*_*i*_ into *Unsafe*_*Sequence*[]

6: **else**

7:  Complete *P*_*i*_ = 0, Push *P*_*i*_ into Ready Queue (RQ)

8:  *Available*[*j*] -= *Allocation*[*i*, *j*]

9:  *Need*[*i*, *j*] ← *Maximum*[*i*, *j*] − *Allocation*[*i*, *j*], *Max*_*Need* ← *max*(*Need*[*i*, *j*])

10:  *SRQ* ← *Sort*(*RQ*)     ⊳ According to *Max*_*Need* in ascending order

11: **end if**

12: **if** Processor == Free && *SRQ* ≠ Empty **then**

13:  Pop the first Process *P*_*i*_ from the *SRQ*, set *k* = 1

14:  **if** *k* ≤ *floor*(*d*/2) **then**

15:   **for** From all unmarked *Available*[*j*] **do**

16:    set *Max*_*Need* ← *Max*(*Need*[*i*, *j*])

17:    set *Min*_*A* ← *Min*(*Available*[*j*])

18:   **end for**

19:   **if** *Max*_*Need* ≤ *Min*_*A* **then**

20:    go to step 42

21:   else

22:    *L*1← Location of *Max*_*Need*

23:    *L*2← Location of *Min*_*A*

24:    **if** (*Need*[*i*, *L*1] > *Available*[*L*1]) || (*Need*[*i*, *L*2] > *Available*[*L*2]) **then**

25:     Push *P*_*i*_ into *Primary*_*Unsafe*_*Sequence*[], go to step 44

26:    **else**

27:     set *Available*[*L*1] &*amp*;&*amp*; *Available*[*L*2] = marked, k++, go to step 14

28:    **end if**

29:   **end if**

30:  **else**

31:   **if** all *Available*[*j*] == marked **then**

32:    go to step 42

33:   **else**

34:    *L*3 ← Location of the unmarked resource

35:    **if** *Need*[*i*, *L*3] > *Available*[*L*3] **then**

36:     Push *P*_*i*_ into *Primary*_*Unsafe*_*Sequence*[], go to step 44

37:    **else**

38:     go to step 42

39:    **end if**

40:   **end if**

41:  **end if**

42:  Complete[*P*_*i*_] = 1, Push *P*_*i*_ into *Safe*_*Sequence*[]

43:  *Available*[*j*] += *Allocation*[*i*, *j*], set all *Available*[*j*] = unmarked

44:  **if** Current_time ≥ *Timer* **then**

45:   return *Safe*_*Sequence*[], *Unsafe*_*Sequence*[], *Primary*_*Unsafe*_*Sequence*[]

46:   Add all Processes in the *Primary*_*Unsafe*_*Sequence*[] into the *SRQ*

47:   go to step 12

48:  **else**

49:   go to step 12

50:  **end if**

51: **else**

52:  Wait

53: **end if**

**Fig 2:**
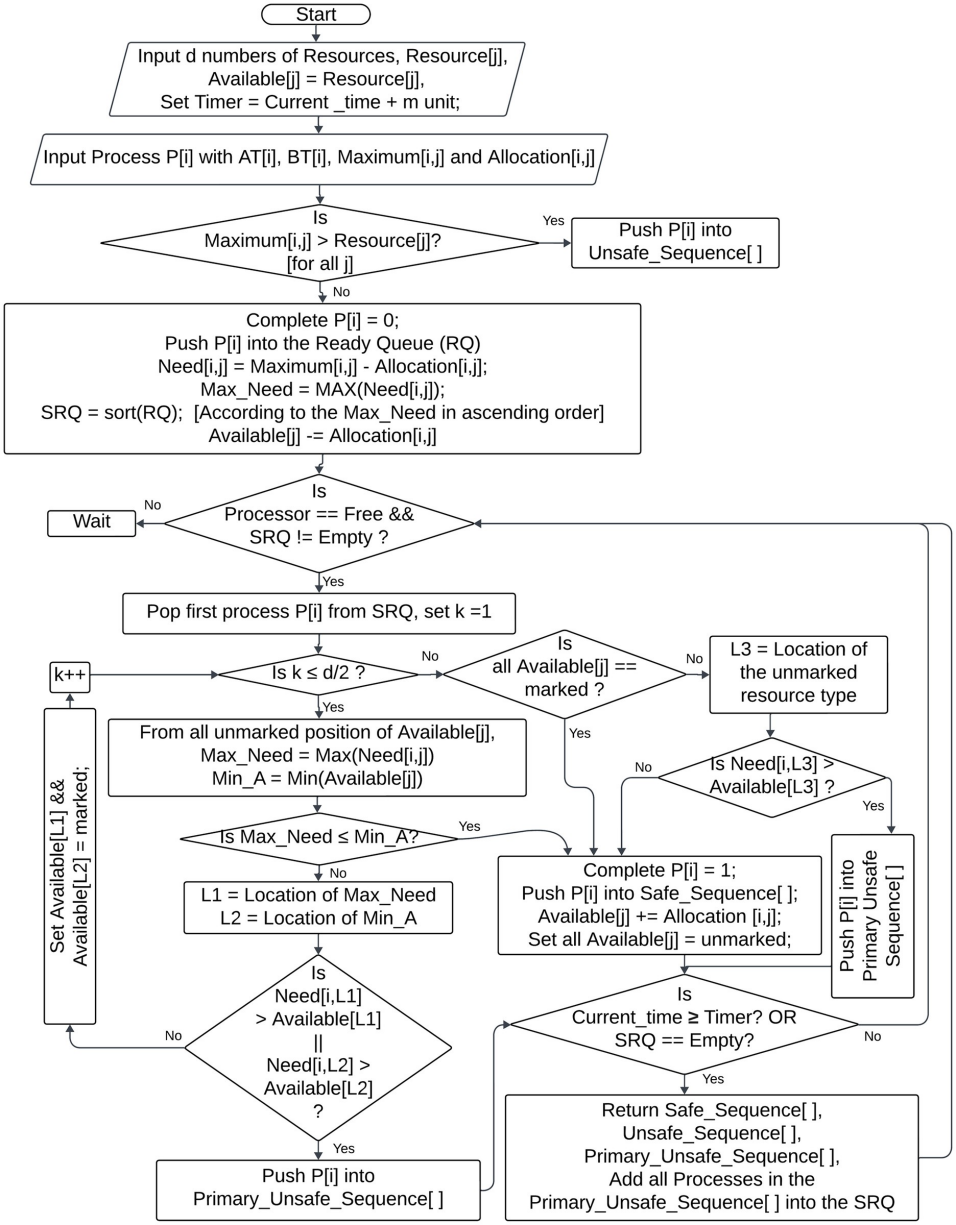
Overview of DBDAA flowchart that depicts the working procedure of the algorithm.

Fig 2 depicted the detailed flow chart of our proposed DBDAA algorithm.

### Experimental data

The total number of resources and the *Need* matrix for each process convey the basis of the experimental analysis. We considered 10 different processes with individual *AT* and *BT* with appropriate Maximum resource requisition capacity and Allocation of resources to explain our designed algorithm for the best case [Table 2], average case [Table 3] and the worst case [Table 4] scenario. For each scenario, there are five different resource types (R1 = 20, R2 = 25, R3 = 30, R4 = 35, and R5 = 30) and a cycle duration of *m*=30 unit. The ID, AT, and BT of each process are represented in the first column of each table; the maximum need of each resource type for a process is indicated in the second column; and the quantity of allocated resources of each kind for each process is stated in the third column. Eq 1 was then used to determine the quantity of additional resources needed by each process which is displayed in the fourth column.

$$\begin{array}{c} Need[i,j]=Maximum[i,j]-Allocation[i,j] \end{array}$$ (1)

**Table 2:** List of processes with initial information for the best case scenario.

Process			Maximum					Allocation					Need				
ID	AT	BT	R1	R2	R3	R4	R5	R1	R2	R3	R4	R5	R1	R2	R3	R4	R5
**P1**	0	3	8	4	11	11	5	4	2	3	4	2	4	2	8	7	3
**P2**	0	4	5	6	4	12	7	0	3	2	5	3	5	3	2	7	4
**P3**	0	2	7	15	12	10	5	2	1	2	1	3	5	14	10	9	2
**P4**	0	1	7	8	5	3	5	1	5	3	2	2	6	3	2	1	3
**P5**	0	5	14	8	7	7	4	3	1	1	3	1	11	7	6	4	3
**P6**	0	3	6	18	10	14	4	1	2	0	2	0	5	16	10	12	4
**P7**	0	2	5	5	4	5	5	3	2	1	3	4	2	3	3	2	1
**P8**	0	4	12	12	15	9	20	2	0	2	4	2	10	12	13	5	18
**P9**	0	1	2	5	4	10	5	0	2	3	5	3	2	3	1	5	2
**P10**	0	5	2	3	3	3	3	0	2	3	2	2	2	1	0	1	1

**Table 3:** List of processes with initial information for average case scenario.

Process			Maximum					Allocation					Need				
ID	AT	BT	R1	R2	R3	R4	R5	R1	R2	R3	R4	R5	R1	R2	R3	R4	R5
**P1**	0	3	9	10	8	12	10	2	4	2	2	2	7	6	6	10	8
**P2**	0	4	10	13	12	14	14	1	3	3	3	4	9	10	9	11	10
**P3**	0	2	12	9	10	9	8	3	1	2	2	2	9	8	8	7	6
**P4**	0	1	3	3	4	4	5	1	1	1	1	0	2	2	3	3	4
**P5**	0	5	5	7	5	7	9	1	2	3	4	3	4	5	2	3	6
**P6**	0	3	6	4	5	6	2	2	1	1	1	1	4	3	4	5	1
**P7**	0	2	16	14	13	17	14	1	2	4	5	4	15	12	9	12	10
**P8**	0	4	13	12	16	20	20	3	0	3	3	5	10	12	13	17	15
**P9**	0	1	12	14	14	13	16	2	2	4	4	3	10	12	10	9	13
**P10**	0	5	15	22	14	15	20	0	4	4	2	0	15	18	10	13	20

**Table 4:** List of processes with initial information for the worst case scenario.

Process			Maximum					Allocation					Need				
ID	AT	BT	R1	R2	R3	R4	R5	R1	R2	R3	R4	R5	R1	R2	R3	R4	R5
**P1**	0	3	15	16	17	19	18	5	4	2	2	4	10	12	15	17	14
**P2**	0	4	8	6	8	11	10	2	2	3	4	2	6	4	5	7	8
**P3**	0	2	5	5	5	8	5	4	3	2	4	3	1	2	3	4	2
**P4**	0	1	9	8	14	18	15	1	1	1	3	2	8	7	13	15	13
**P5**	0	5	16	19	22	30	24	0	2	2	4	0	16	17	20	26	24
**P6**	0	3	15	18	23	25	26	0	2	3	2	4	15	16	20	23	22
**P7**	0	2	6	7	10	12	11	1	1	1	3	3	5	6	9	9	8
**P8**	0	4	19	20	27	28	26	2	3	4	3	2	17	17	23	25	24
**P9**	0	1	10	12	13	16	17	1	4	3	0	3	9	8	10	16	14
**P10**	0	5	17	17	19	26	22	2	1	2	2	2	15	16	17	24	20

The analysis was carried out under the assumptions of a single processor environment, considering burst times before execution, and non-consequential sorting time.

## Results and discussion

The overall process of the proposed DBDAA has been mathematically explained here. Our algorithm was also compared with five other safety detection methods for validation.

### Experimental results

The determination of the *Safe*_*Sequence*, *Primary*_*Unsafe*_*Sequence*, and *Unsafe*_*Sequence* with the required number of comparisons within a certain time frame (*m*) has been described here. Intel Core i7 processor with 16 GB of RAM and 4GB Nvidia 920MX GPU were used in the preliminary test. The experiment was carried out using 3 types of datasets shown in [Tables 2–4] all having a zero *AT* and random *BT*.

#### Case 1: Best case

In Table 2, we considered 10 individual processes to evaluate our algorithm with random *BT*. In this case, all the processes are assumed to arrive at the system at *AT*=0 for simplification. To identify the safe sequence, we must first determine each process’s Maximum Need as *Max*_*Need*. We create the *SRQ* of 10 processes based on their *Max*_*Need* in ascending order, as shown in the first column of Table 5. *P*10 is consequently positioned at the top of the *SRQ*, whereas *P*8 is positioned at the bottom. Before initiating the safety check method, we use Eq 2 to determine the number of available resources in the system in the third column. In this example, resources R1 = 4, R2 = 5, R3 = 10, R4 = 4, and R5 = 8 are available before starting the safety check algorithm. From the currently available resources [4, 5, 10, 4, 8] we calculate the minimum of them as *Min*_*A* which is 4, and put it on the fourth column of Table 5.

$$\begin{array}{c} Available\left[j\right]=Available\left[j\right]-\sum \left(Allocation\left[i,j\right]\right) \end{array}$$ (2)

**Table 5:** Best case example, processes are sorted in ascending order according to their maximum need.

Process	Maximum Need		Available					Minimum Available		Is safe to Execute?	No. of Comparison
	1st	2nd	R1	R2	R3	R4	R5	1st	2nd		
**P10**	2		4	5	10	4	8	4		**Yes**	**1**
**P7**	3		4	7	13	6	10	4		**Yes**	**1**
**P9**	5		7	9	14	9	14	7		**Yes**	**1**
**P4**	6		7	11	17	14	17	7		**Yes**	**1**
**P2**	7		8	16	20	16	19	8		**Yes**	**1**
**P1**	8		8	19	22	21	22	8		**Yes**	**1**
**P5**	11		12	21	25	25	24	12		**Yes**	**1**
**P3**	14		15	22	26	28	25	15		**Yes**	**1**
**P6**	16		17	23	28	29	28	17		**Yes**	**1**
**P8**	18		18	25	28	31	28	18		**Yes**	**1**

Now for P10, we check if the *Max*_*Need* ≤ *Min*_*A* ensures that the system has all types of resources available as required by the considered process. So we get it true and the process has completed its safety check in one comparison. Now the available resources are [R1, R2, R3, R4, R5]=[4, 7, 13, 6, 10] and their Minimum Available, *Min*_*A* is 4.

Subsequently, we determine if the remaining processes (*P*7, *P*9, *P*4, *P*2, *P*1, *P*5, *P*3, *P*6, and *P*8) in the *SRQ* are qualified to maintain system safety. We found that all of them fulfill the criteria “*Max*_*Need* ≤ *Min*_*A*” ensuring that they need only one comparison per process to decide the safe state of the system correctly.

**Fig 3:**
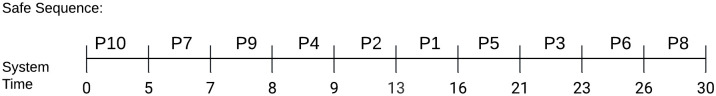
Gantt chart for the best-case process execution request.

The Gantt chart in Fig 3 depicts the *Safe*_*Sequence* of 10 processes with the system time. Since our cycle length was initialized as *m*=30 unit, the safety check method began at 0 and ended at 30 for one episode. Within the specified time frame, we observed that no processes were in either the *Unsafe*_*Sequence* or *Primary*_*Unsafe*_*Sequence*.

#### Case 2: Average case

In Table 3, we considered 10 individual processes to evaluate our algorithm with random *BT*. To keep operations simple, it is assumed that every process will arrive at the system at *AT*=0. We need to determine the Maximum Need as *Max*_*Need* for each process before identifying the safe sequence. As indicated in the first column of Table 6, we create the *SRQ* of 10 processes based on their *Max*_*Need* in ascending order. As a result, *P*10 is placed at the bottom of the *SRQ*, and *P*4 is at the top. Before initiating the safety check method, we use Eq 2 to determine the number of available resources in the system in the third column. In this example, resources R1 = 4, R2 = 5, R3 = 3, R4 = 8, and R5 = 6 are available before starting the safety check algorithm. From the current available resources [4, 5, 3, 8, 6] we calculate the minimum of them as *Min*_*A* which is 3, and put it in the fourth column of Table 6.

**Table 6:** Average case example, processes are sorted in ascending order according to the maximum need.

Process	Maximum Need		Available					Minimum Available		Is safe to Execute?	No. of Comparison
	1st	2nd	R1	R2	R3	R4	R5	1st	2nd		
**P4**	4	3	4	5	3	8	6	3	4	**Yes**	**4**
**P6**	5	4	5	6	4	9	6	4	5	**Yes**	**4**
**P5**	6	5	7	7	5	10	7	5	7	**Yes**	**4**
**P3**	9		8	9	8	14	10	8		**No**	**2**
**P1**	10	8	8	9	8	14	10	8	8	**Yes**	**4**
**P2**	11	10	10	13	10	16	12	10	10	**Yes**	**4**
**P9**	13	12	11	16	13	19	16	11	13	**Yes**	**4**
**P7**	15		13	18	17	23	19	13		**No**	**2**
**P8**	17	15	13	18	17	23	19	13	17	**Yes**	**4**
**P10**	20	18	16	18	20	26	24	16	18	**Yes**	**4**

Now we pop the first process, *P*4, from the *SRQ* and check if the *Max*_*Need* ≤ *Min*_*A* ensures that the system has all types of resources available as required by the considered process. But we get it false for *P*4 as 4 > 3. According to our algorithm, we find the position of the *Max*_*Need* (5) and the *Min*_*A* (3). Next, we determine if *Need*[*P*4, *R*5] ≤ *Available*[*R*5] and *Need*[*P*4, *R*3] ≤ *Available*[*R*3]. We get it true as 4 ≤ 6 and 3 ≤ 3. At this point, we have ensured that resource R3 and resource R5 of P4 are available in the system to execute and keep them as marked. The availability of the remaining three resources (R1, R2, and R4) must now be confirmed. For this reason, we find the *Max*_*Need*(3) of *P*4 and the *Min*_*A*(4) from the unmarked resource set. We now recheck the “*Max*_*Need* ≤ *Min*_*A*” criterion for *P*4 and find that it is true as 3≤4, indicating that *P*4 has all the resources it needs accessible in the system. In this case, our method required four comparisons to complete the *P*4 safety check.

Comparably, we determine if the remaining processes in the *SRQ* (*P*6, *P*5, *P*3, *P*1, *P*2, *P*9, *P*7, *P*8, and *P*10) are qualified to maintain the system in a safe state or not. Except for *P*3 and *P*7, we found that each one needed four comparisons to determine the system’s safe state. We observed that *Need*[*P*3, *R*1] > *Available*[*R*1] and *Need*[*P*7, *R*1] > *Available*[*R*1] in the second comparison. Consequently, these two processes are added to the *Primary*_*Unsafe*_*Sequence* since they are deemed unsafe for that time slice. All the processes in the *Primary*_*Unsafe*_*Sequence* will be restored into the *SRQ* for a safety check immediately after one cycle (*m*) has been completed.

**Fig 4:**
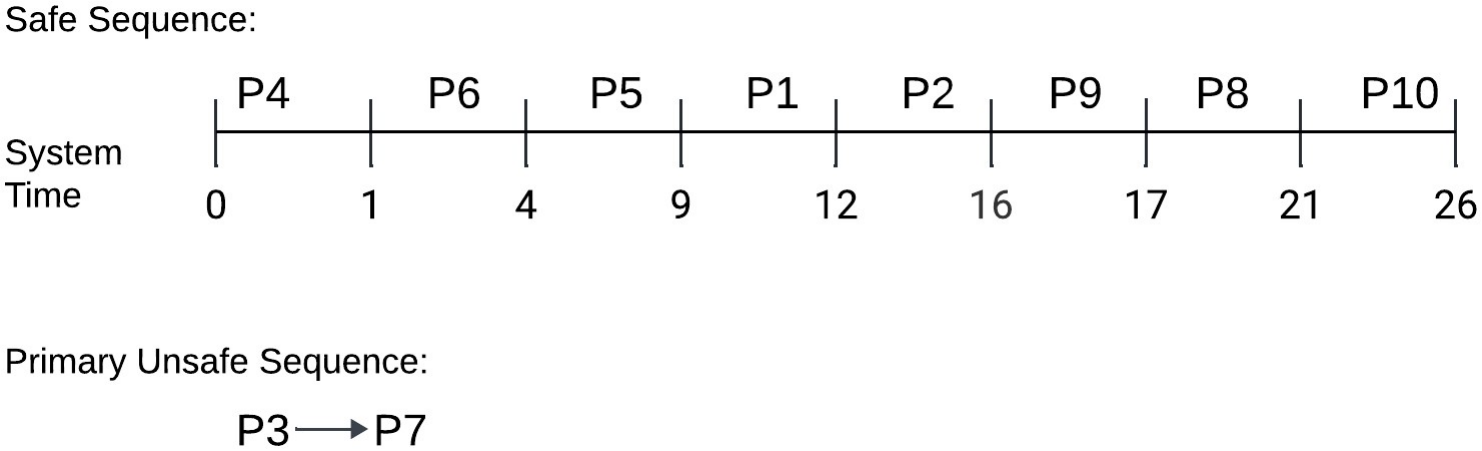
Gantt chart for the average-case process execution request.

The Gantt chart in Fig 4 depicts the *Safe*_*Sequence* of 8 processes within the system time. We started with a cycle length of *m*=30. The safety check method began at 0 and ended at 26 for one episode as the *SRQ* was empty. We saw that processes *P*3 and *P*7 were in the *Primary*_*Unsafe*_*Sequence* within the allotted time.

#### Case 3: Worst case

In Table 4, we considered 10 individual processes to evaluate our algorithm with random *BT*. To keep operations simple, in this case, it is assumed that every process will arrive at the system at *AT*=0. We need to determine the Maximum Need Allocation as *Max*_*Need* for each process before identifying the safe sequence. As indicated in the first column of Table 7, we create the *SRQ* of 10 processes based on their *Max*_*Need* in ascending order. As a result, *P*3 is placed at the top and *P*5 is at the bottom of the *SRQ*. Before initiating the safety check method, we use Eq 2 to determine the number of available resources in the system in the third column. In this example, resources R1 = 2, R2 = 2, R3 = 7, R4 = 8, and R5 = 5 are available before starting the safety check algorithm. From the currently available resources [2, 2, 7, 8, 5] we calculate the minimum of them as *Min*_*A* which is 2 and put it in the fourth column of Table 7.

**Table 7:** Worst case example, processes are sorted in ascending order according to the maximum need.

Process	Maximum Need		Available					Minimum Available		Is safe to Execute?	No. of Comparison
	1st	2nd	R1	R2	R3	R4	R5	1st	2nd		
**P3**	4	3	2	2	7	8	5	2	2	**Yes**	**7**
**P2**	8	7	6	5	9	12	8	5	6	**Yes**	**7**
**P7**	9	9	8	7	12	16	10	7	8	**Yes**	**7**
**P4**	15	13	9	8	13	19	13	8	9	**Yes**	**7**
**P9**	16	14	10	9	14	22	15	9	10	**Yes**	**7**
**P1**	17	15	11	13	17	22	18	11	13	**Yes**	**7**
**P6**	23	22	16	17	19	24	22	16	17	**No**	**7**
**P10**	24	20	16	17	19	24	22	16	17	**Yes**	**7**
**P8**	25	24	18	18	21	26	24	18	18	**No**	**7**
**P5**	26	24	18	18	21	26	24	18	18	**Yes**	**7**

Now we pop the first process, *P*3, from the *SRQ* and check if the *Max*_*Need* ≤ *Min*_*A* ensures that the system has all types of resources available as required by the considered process. But we get it false for *P*3 as 4 > 2. According to our algorithm, we find the position of the *Max*_*Need*(4) and the *Min*_*A* (1). Next, we determine if *Need*[*P*3, *R*4] ≤ *Available*[*R*4] and *Need*[*P*3, *R*1] ≤ *Available*[*R*1]. We get it true as 4≤8 and 1≤2. At this point, we have ensured that resource R1 and resource R4 of *P*3 are available in the system to execute and keep them as marked. Verifying the availability of the remaining three resources (R2, R3, and R5) is now required. As a result, we determine *P*3’s *Max*_*Need* (3) and *Min*_*A* (2) from the resource set that is not marked. The “*Max*_*Need* ≤ *Min*_*A*” condition for *P*3 is now verified again, and we discover that 3 > 2. Thus, applying our method, we find the position of the *Max*_*Need* (3) and the *Min*_*A* (2). We then verify whether *Need*[*P*3, *R*3] ≤ *Available*[*R*3] and *Need*[*P*3, *R*2] ≤ *Available*[*R*2]. It turns out to be true as 2≤2 and 3≤7. Thus far, we have confirmed that *P*3’s resources R1, R2, R3, and R4 are available within the system for use and keep them as marked. There is now just one resource (R5) left to verify, and after checking this directly, we discovered that the system has enough R5 available to execute *P*3. In this instance, completing the *P*3 safety check with our method required seven comparisons.

Subsequently, we determine if the remaining processes (*P*2, *P*7, *P*4, *P*9, *P*1, *P*6, *P*10, *P*8, and *P*5) in the *SRQ* are qualified to maintain system safety. We discovered that each process required seven comparisons, except *P*6 and *P*8 to identify the system’s safe state. We observed that *Need*[*P*6, *R*3] > *Available*[*R*3] and *Need*[*P*8, *R*3] > *Available*[*R*3] in the seventh comparison. Consequently, these two processes are added to the *Primary*_*Unsafe*_*Sequence* since they are deemed unsafe for that time slice. All of the processes in the *Primary*_*Unsafe*_*Sequence* will be restored into the *SRQ* for safety check immediately after one cycle is completed.

**Fig 5:**
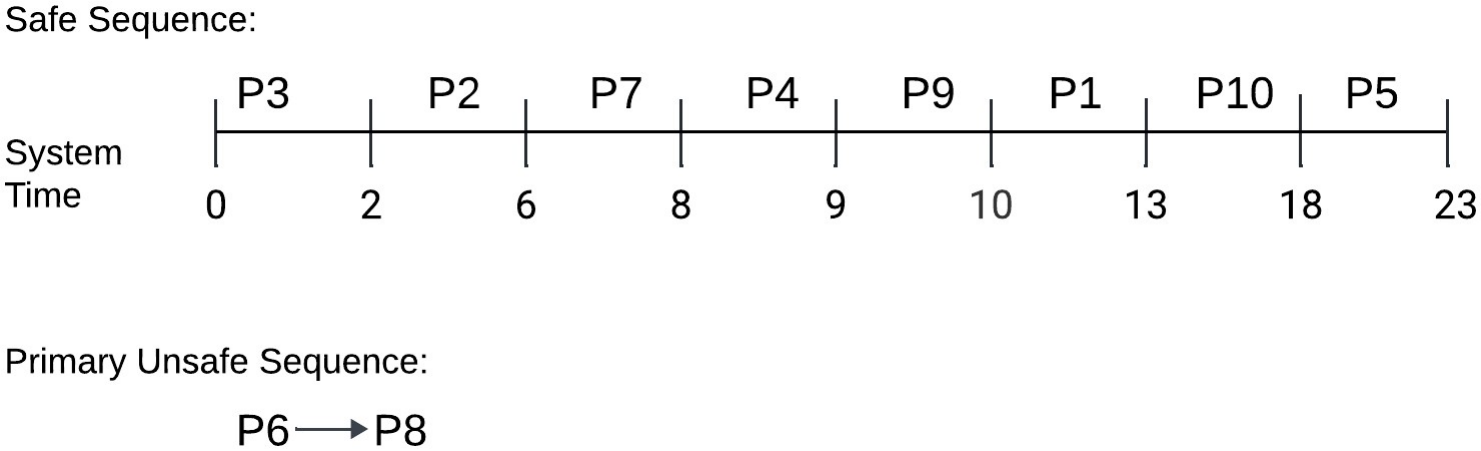
Gantt chart for the worst-case process execution request.

The Gantt chart in Fig 5 depicts the Safe Sequence of 8 processes within the system time. We started with a cycle length of *m*=30. The safety check method began at 0 and ended at 23 for one episode as the *SRQ* was empty. We saw that processes *P*6 and *P*8 were in the *Primary*_*Unsafe*_*Sequence* within the allotted time.

### Result comparison and discussion

We compared the proposed safety detection algorithm to five different methods, including the original banker’s algorithm, to validate the efficiency of the suggested method. Table 8 display the results of the comparison. We utilized the identical dataset from Tables 2–4 to compare various algorithms.

**Table 8:** Number of Comparisons required by different safety algorithms.

Different Algorithms	Original Banker’s Algorithm [1]	Momotaz *et al*.,2020 [4]	Bondarenko *et al*.,2021 [5]	Song *et al*.,2021 [9]	Wicaksono *et al*.,2023 [12]	DBDAA (This study)
**Dataset**: Table 2	85	10	85	85	85	10
**Dataset**: Table 3	100	Unsafe	100	100	100	36
**Dataset**: Table 4	125	Unsafe	125	125	125	70

For the dataset from Table 2, we can see that both DBDAA and Momotaz *et al*. [4] performed the safety check using 10 comparisons, and the resultant safe sequence is < *P*10, *P*7, *P*9, *P*4, *P*2, *P*1, *P*5, *P*3, *P*6, *P*8 >. The remaining four methods, [1, 5, 9, 12] determine the safe sequence < *P*7, *P*1, *P*2, *P*4, *P*3, *P*5, *P*6, *P*8, *P*9, *P*10 > by employing 85 comparisons.

Our approach required 36 comparisons to produce two sequences for the dataset from Table 3: the *Safe*_*Sequence* <*P*4, *P*6, *P*5, *P*1, *P*2, *P*9, *P*8, *P*10> and the *Primary*_*Unsafe*_*Sequence* <*P*3, *P*7>. The processes in the *Primary*_*Unsafe*_*Sequence* are added back in the *SRQ* for a safety check after completion of one cycle (*m*). However, to produce the safe sequence <*P*4, *P*5, *P*6, *P*1, *P*2, *P*3, *P*8, *P*7, *P*9, *P*10>, the algorithms [1, 5, 9, 12] required 100 comparisons. Momotaz *et al*. [4] could not determine the safe sequence in this dataset and instead predicted that the system was in an “Unsafe” state which is not true.

For the dataset from Table 4, our method produced two sequences; The *Safe*_*Sequence*: < *P*3, *P*2, *P*7, *P*4, *P*9, *P*1, *P*10, *P*5 > and the *Primary*_*Unsafe*_*Sequence*: < *P*6, *P*8 >. This required 70 comparisons. After one cycle (*m*), the processes in the *Primary*_*Unsafe*_*Sequence* are added back into the *SRQ* for safety check again. However, 125 comparisons were needed by the algorithms [1, 5, 9, 12] in order to generate the safe sequence < *P*3, *P*2, *P*7, *P*4, *P*9, *P*1, *P*10, *P*5, *P*6, *P*8 >. Momotaz *et al*. [4] projected that the system was in an “unsafe” state which is an error because it could not identify the safe sequence in this dataset.

**Fig 6:**
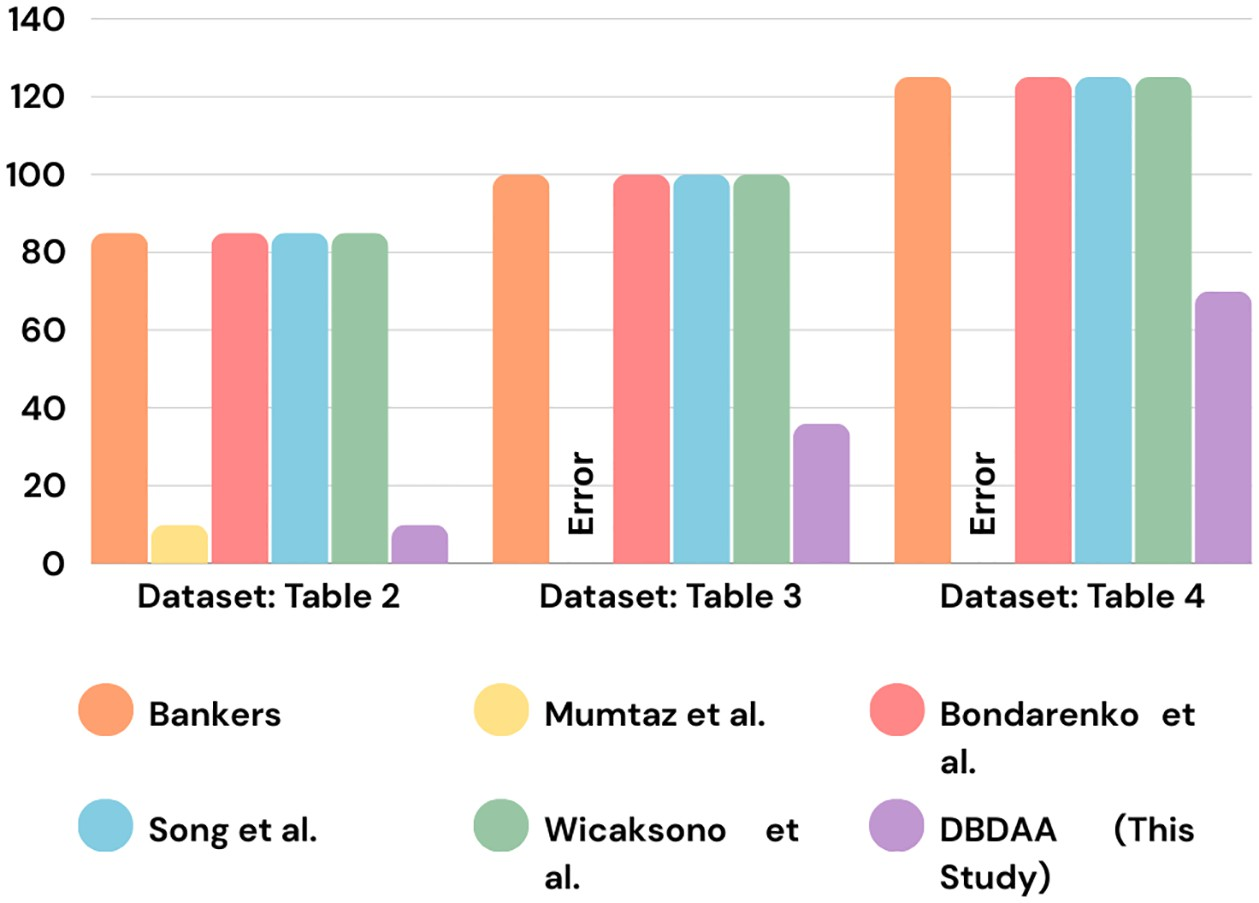
Comparative analysis of the number of comparisons required by DBDAA and other traditional safety detection algorithms.

The analysis shows that the proposed DBDAA outperforms other popular safety check methods by reducing the number of comparisons with accuracy. Fig 6 represents a comparative analysis of the number of comparisons required by DBDAA and other traditional safety detection algorithms.

#### Time complexity analysis

In the Methodology section of DBDAA, we can see that, step 6 (Safety Check) is the core part of the algorithm that uses several comparisons on which the time complexity of the procedure depends. In the best-case scenario, it takes *O*(*n*) times, regardless of the quantity of resource types (*d*). Our simplified average and worst-case time complexity is *O*(*nd*).

**Fig 7:**
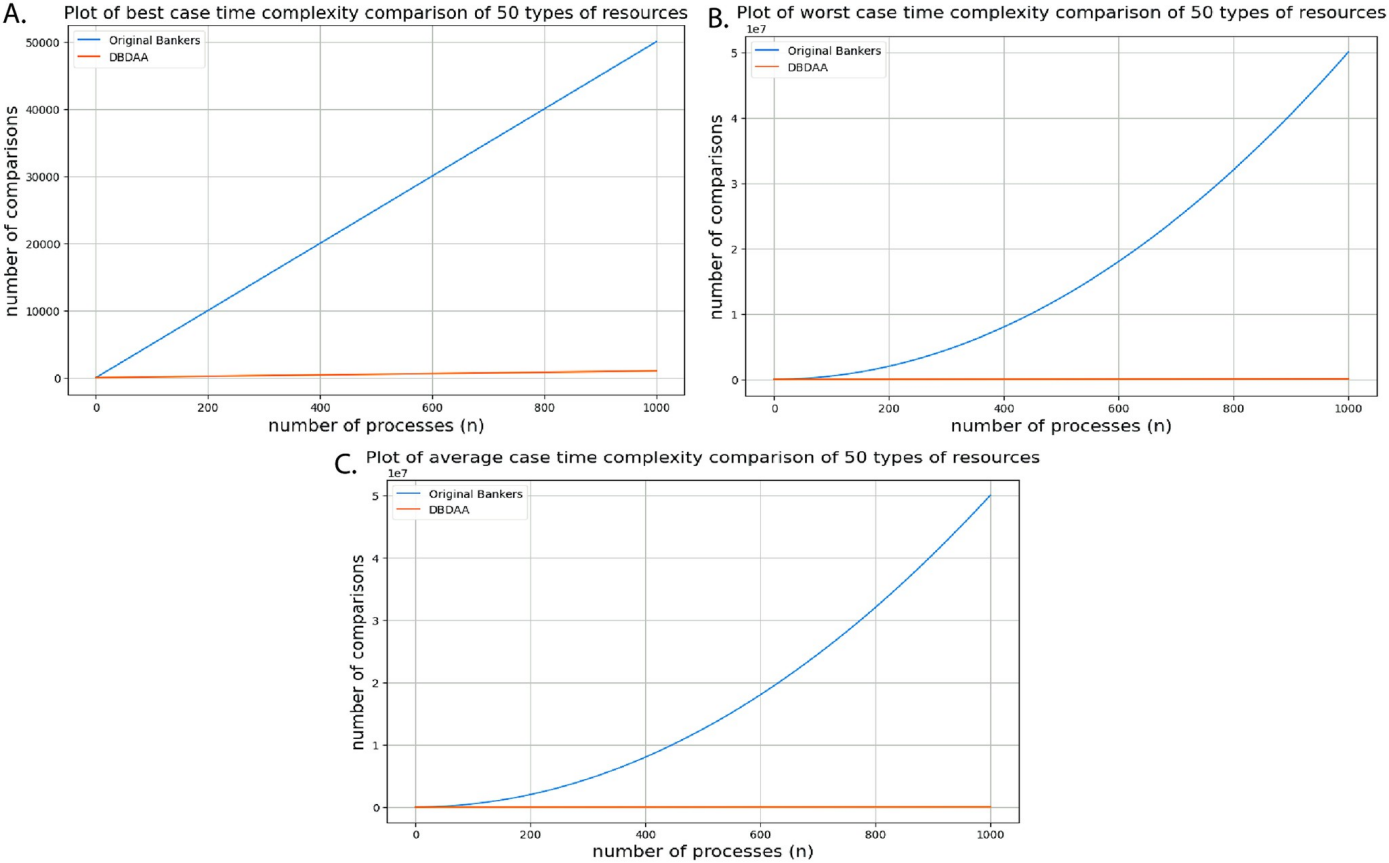
Comparison of Time Complexity between the Traditional Banker’s Algorithm and the Proposed DBDAA under A. Best case B. Worst case C. Average case Scenario.

Table 9 represents the time complexity of different safety algorithms for their best-case, average-case, and worst-case scenarios. A comparison of time complexity between the traditional Banker’s algorithm and the proposed DBDAA under A. Best case B. Worst case C. Average case scenario is illustrated in Fig 7.

**Table 9:** Time complexity of different safety algorithms.

Algorithm	Time Complexity		
	Best case	Average case	Worst case
Original Banker’s Algorithm [1]	*O*(*nd*)	*O*(*n*^2^*d*)	*O*(*n*^2^*d*)
Momotaz *et al* [4]	*O*(*n*)	*O*(*nd*)	*O*(*n*(*d* + 2))
Bondarenko *et al* [5]	*O*(*nd*)	*O*(*n*^2^*d*)	*O*(*n*^2^*d*)
Song *et al* [9]	*O*(*nd*)	*O*(*n*^2^*d*)	*O*(*n*^2^*d*)
Wicaksono *et al* [12]	*O*(*nd*)	*O*(*n*^2^*d*)	*O*(*n*^2^*d*)
DBDAA	*O*(*n*)	*O*(*n*(3*d* + 2)/4)	*O*(*n*(*d* + *d*/2))

## Conclusion and future direction

In this paper, we suggested a new method based on the well-known Banker’s algorithm to dynamically identify the safe sequence of processes within a certain period. Research and testing demonstrated that our proposed approach produced better outcomes with more efficient time complexities when compared to the original Banker’s method and other existing safety detection techniques. Our technique used fewer comparisons for large dimensional permutation matrices. Real-time processes can enter the system and take part in the safety testing procedure instantly. For a certain number of resources in the system, we identify the sequence of the safe processes, primarily unsafe processes and completely unsafe processes within a given time frame. The primary unsafe processes are assessed again through the proposed safety detection algorithm in the next period so that each eligible process can get a chance more than once. We want to use the newly proposed approach in the future to prioritize processes, prevent deadlocks, and carry out a more thorough study to verify our approach with a few inappropriate processes.

## Supporting information

S1 File Data availability statement of DBDAA. (DOCX)
