# Supplementary material for: DBDAA: A real-time approach to Dynamic Banker’s Deadlock Avoidance Algorithm with optimized time complexity
Source: PLoS One. 2024 Sep 20;19(9):e0310807. doi: 10.1371/journal.pone.0310807 (PMC11414889; doi:10.1371/journal.pone.0310807)

We considered 10 different processes with individual Arrival Time and Burst Time with appropriate Maximum resource requisition capacity and Allocation of resources to explain our designed algorithm for the best case [Table 2], average case [Table 3] and the worst case [Table 4] scenario. For each scenario, there are five different resource types (R1=20, R2=25, R3=30, R4=35, and R5=30) and a cycle duration of m=30 unit. The ID, Arrival Time, and Burst Time of each process are represented in the first column of each table; the maximum need of each resource type for a process is indicated in the second column; and the quantity of allocated resources of each kind for each process is stated in the third column. Equation 1 was then used to determine the quantity of additional resources needed by each process which is displayed in the fourth column.

Need[i,j] = Maximum[i,j] - Allocation[i,j] ….. [Equation 1]

The analysis was carried out under the assumptions of a single processor environment, considering burst times before execution, and non-consequential sorting time. For comparing our model with other existing models we used the same 3 dataset.


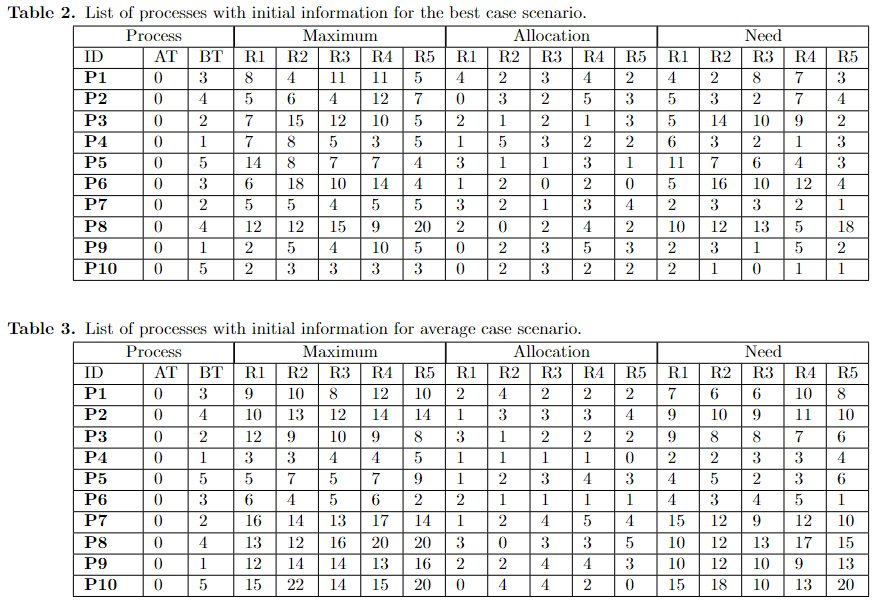


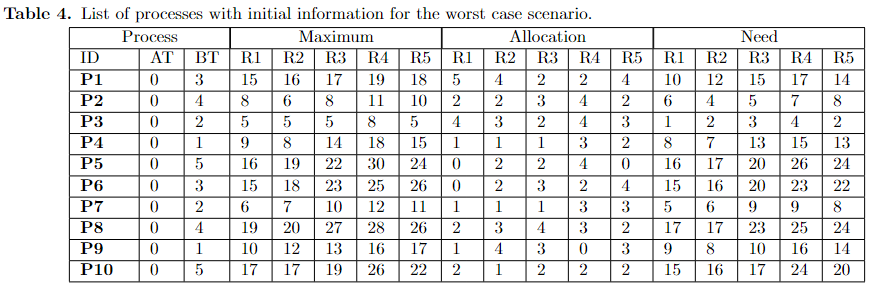

Supplement: S1 File — (DOCX) [file pone.0310807.s001.docx]
